# Visitor characteristics and changes in mental health stigma after attending the Mind Space mental health experience exhibition

**DOI:** 10.3389/fpsyt.2024.1302799

**Published:** 2024-04-29

**Authors:** Grace W. K. Ho, Jolene Hang Chun Mui, Raymond Wong, Wai Tong Chien, Kwan Ho Wong, Daniel Bressington

**Affiliations:** ^1^ School of Nursing, Hong Kong Polytechnic University, Hong Kong, Hong Kong SAR, China; ^2^ School of Nursing and Health Studies, Hong Kong Metropolitan University, Hong Kong, Hong Kong SAR, China; ^3^ Castle Peak Hospital, Hong Kong, Hong Kong SAR, China; ^4^ The Nethersole School of Nursing, The Chinese University of Hong Kong, Hong Kong, Hong Kong SAR, China; ^5^ College of Nursing and Midwifery, Charles Darwin University, Darwin, NT, Australia

**Keywords:** mental health, mental health literacy, services evaluation, mental health exhibition, stigma, mental health education, public mental health

## Abstract

**Introduction:**

Mind Space is an experiential mental health exhibition in Hong Kong, aiming to raise public awareness and provide education regarding mental health. This prepost study aimed to 1) examine the relationships between visitors’ characteristics and their mental health stigma at baseline, and 2) provide a preliminary evaluation of the effectiveness of Mind Space in reducing stigma and promoting help-seeking attitudes toward mental health conditions.

**Methods:**

We analyzed data from all consenting visitors who attended Mind Space between September 2019 and December 2021. Visitors’ attitudes toward mental health conditions and their willingness to seek professional psychological help were measured through online questionnaires before and after visits. Multiple linear regression was used to identify the demographic predictors of outcome variables at baseline. Changes in outcome variables after attending Mind Space were assessed using paired sample t-tests.

**Results:**

A total of 382 visitors completed the baseline questionnaires, among which 146 also completed the post-test. At baseline, higher socioeconomic levels and personal contact with people with mental health conditions predicted more positive attitudes and understanding toward mental disorders. Tentatively, the results also showed that after attending Mind Space, a significant reduction in negative attitudes about mental illness (t=4.36, p=<.001; d=.361) and improvements in the propensity to seek professional help (t=-5.20, p<.001; d=-.430) were observed, along with decreases in negative attitudes toward stereotypes (t=4.71, p=<.001; d=.421) and restrictions (t=2.29, p=.024; d=.205) among healthcare professionals.

**Discussion:**

Our findings highlight the need for mental health education for people with lower socioeconomic status and the importance of direct contact in public mental health education initiatives. The present study also suggests that Mind Space may be a useful model for public mental health education, but the exhibition requires further evaluation to ascertain if any reductions in stigma are maintained over time.

## Introduction

It is estimated that one in six Hong Kong adults have a diagnosable mental health condition, and the number of young people referred to child and adolescent psychiatric teams continues to rise (1). Despite the increasing number of people identified as having a mental health condition in Hong Kong, negative attitudes toward mental health conditions are relatively commonplace (2–4) and many people delay seeking treatment as a result (5). These delays are in part associated with low levels of mental health literacy, which has been defined as knowledge and beliefs about mental health conditions that may influence their recognition, management, and prevention (6). Low levels of mental health literacy, including stigmatized beliefs and poor understanding of mental health conditions, are therefore major barriers to receiving prompt mental health treatments that facilitate recovery for people experiencing mental disturbances (7).

Public beliefs and attitudes about mental health conditions can have profound negative impacts on individuals with a mental health condition. Previous studies have showed that negative attitudes and social stigma lead to discriminatory behavior and ultimately the isolation of people who experience mental health conditions (8). Experiencing discrimination is extremely damaging for people with mental health conditions, for example some studies have shown a clear link between suicidality and discrimination (9, 10). The fear of discrimination and negative expectations of recovery fueled by social stigma can also cause people with mental health conditions to develop self-stigma through internalization of these stereotypes, thereby creating a vicious cycle of low self-worth and shame (8). Demoralization is associated with repeated failure and low self-esteem and may also arise due to self-stigmatization; this has also been identified as an independent risk factor for suicide (11).

The problem of self-stigma may also be magnified in Hong Kong because Chinese people are likely to internalize public stigma (12–15). Specifically, Chinese culture, heavily rooted in Confucianism and collectivism, has traditionally emphasized group cohesion, social identity, and maintaining “face” (12, 16). In this context, individuals with mental health conditions are often viewed as deviating from societal norms and failing to fulfill social role expectations, leading to stronger discrimination, heightened stigma, and losing face (15, 17). These experiences may further enhance their internalized stigma (18).

Furthermore, people with mental health conditions in the collectivism culture may be particularly sensitive to public stigma and are more likely to internalize public stigma (14, 15, 18). Indeed, a local study of 311 people with severe mental illness found high levels of self-stigma, secrecy, and withdrawal behaviors (2). The emphasis on “face” concerns in Chinese culture may also impede individuals from seeking professional help because they may avoid the risk of disclosing their mental health problems to avoid shaming the reputation of their own family (17). Consequently, they perceive the need to keep their mental health conditions secret (19, 20), which decreases their likelihood of seeking help (17). Indeed, previous studies have found that face concern was positively associated with self-stigma and public stigma, and both stigma and face concern were negatively related to help-seeking attitudes (17, 21).

Unfortunately, negative views about mental health conditions are also evident in professionals that are responsible for the care of people experiencing mental health conditions; over half of 76 Hong Kong mental health professionals surveyed believed that people with severe mental illness were dangerous, abnormal, and unpredictable (2). It is therefore of paramount importance to improve the mental health related attitudes of a wide range of different people in Hong Kong, from laymen to professionals, in order to reduce the direct and indirect impacts of poor mental health literacy on society.

It is also essential to identify demographic correlates of mental health stigma in the local context to inform targeted recruitment and education for high-risk groups. However, demographic predictors of attitudes toward mental health conditions and its treatment are equivocal in Hong Kong studies. Currently, the majority of existing evidence indicates that women (22–27), people from higher socioeconomic groups (28) and those with a university education (29–31) are most likely to have positive attitudes toward people with mental health conditions and seeking professional psychological help, particularly in studies conducted in Western settings/cultures. However, in contrast with studies conducted in non-Chinese populations, some local research showed that female gender is associated with increased levels of public stigma among Hong Kong residents (32). Another random telephone survey of Hong Kong residents also reported that female gender, better knowledge about symptoms, and higher educational level was associated with more negative attitudes toward psychosis (3).

Numerous studies have focused on increasing the public knowledge of mental health and disorders to reduce the stigma and discrimination associated with mental health conditions. These studies tend to use a variety of strategies, including targeted educational programs, psychoeducational interventions, facilitating direct and indirect contact with someone with a mental health conditions, and mass media information campaigns. Mass media campaigns in England, United States, and India resulted in significant changes in knowledge and attitudes toward mental health conditions in members of the public (33–35) and population-level awareness campaigns in Austria and Germany produced moderate effects on attitudes, but not on knowledge (37). Similarly, the UK’s “Time to Change” media campaign conducted over 3 years resulted in improvements in intended behavior toward seeking treatment and attitudes toward mental health conditions, but no significant changes in knowledge (36).

Given the different outcomes reported from intervention studies, several systematic reviews have considered which specific contents/approaches of anti-stigma interventions are most effective for different demographic groups. The consolidated evidence from such reviews (37, 38) showed social contact with someone with a mental health condition appears to be the most effective approach to improve stigma-related attitudes over the short term across all demographic groups, but the effects are only sustained in the medium term for around 50% of studies. More recently, a scoping review of 16 studies (39) using augmented and virtual reality as an interactive and experiential psychoeducation tool supported their use in improving mental health knowledge, attitudes, empathy, and stigma, but 15 these studies were conducted in Western settings. However, a recent study of university students in Hong Kong showed immersive virtual reality is an effective tool in reducing public stigma toward mental health conditions (40). Overall, this body of evidence highlights more research is needed to understand the needs and effects of different interventions for different populations and in different cultural contexts. A multi-component approach incorporating different evidence-based strategies may also enhance the effectiveness of initiatives aimed to reduce mental health stigma.

In Hong Kong, research has demonstrated that better knowledge about mental health conditions and more positive contact with persons with mental illness were associated with lower stigma (41, 42), but it remains unclear whether sub-groups of Hong Kong citizens are more likely to hold negative stigmatized attitudes and require additional support to improve their attitudes toward seeking professional help.

In response, the Mind Space exhibition at Castle Peak Hospital was established to provide a permanent interactive mental health learning experience to provide mental health education and reduce stigma for members of the public from different backgrounds using a variety of evidence-based strategies. The overall objectives of Mind Space are to raise public awareness and provide education regarding mental health; reduce the stigma of mental health conditions so that those suffering from mental illness may seek help early; and provide a genuinely interactive, meaningful, and memorable mental health educational experience. The multi-component approach of Mind Space, including individual exhibits, experience-based learning activities, interactive displays, virtual reality simulations, and facilitated contact with people with lived experience of mental illness, were designed based on, and consolidates, the evidence of effectiveness from previous public mental health education initiatives.

### The present study

The aim of this study was to provide a preliminary evaluation of Mind Space using pre- and post-test data collected from its visitors since the exhibition was opened in September 2019. Specifically, baseline mental health stigma and the effects of attending the exhibition experience on visitors’ attitudes toward mental health conditions and attitudes toward seeking professional mental health care before and after the visit were assessed.

The specific objectives of this evaluation study were to: (1) Describe the characteristics of visitors at Mind Space and examine demographic correlates associated with the visitors’ attitudes and understanding toward mental health conditions, and their attitudes toward seeking professional psychological help before attending Mind Space; and (2) provide a preliminary evaluation of changes in visitors’ attitudes and understanding toward mental health conditions, and their attitudes toward seeking professional psychological help before attending Mind Space and immediately following their visit.

Our main study hypothesis, based on the extant evidence, was that attending the exhibition and engaging with its interactive experiential learning activities would result in positive changes in visitors’ attitudes and understanding toward mental health conditions, and their attitudes toward seeking professional psychological help. We also hypothesized that male gender, higher social economic groups, university education and previous contact with a person with mental health conditions would be associated with more positive understanding and attitudes toward mental health conditions and attitudes toward seeking professional psychological help.

## Methods

This evaluation study was conducted at the Mind Space mental health experience exhibition at Castle Peak Hospital, Hong Kong. It is located at the education and training building within the hospital grounds. We adopted a simple pre-post study design rather than a controlled experimental design to evaluate changes in visitors’ attitudes because the Mind Space exhibition was newly opened to the public and it was not feasible to establish a control condition (i.e. random allocation to a wait-list control group or similar). We analyzed data from all consenting visitors who attended Mind Space and provided pre-visit survey data between September 2019 and December 2021.

All visitors were asked to register their visits and complete a set of online questionnaires before and immediately after their visits. Ethical approvals to conduct the evaluation were obtained from the first author’s institution and the Hong Kong Hospital Authority Cluster Clinical Research Ethics Committee.

### Mind Space

The Mind Space exhibition was developed on the site of the previous Castle Peak Hospital Archive. Castle Peak Hospital is the oldest and perhaps the most well-known psychiatric hospital in Hong Kong (43). On arrival to Mind Space, visitors enter through a large gate that is a reproduction of the original entrance to the hospital. Visitors are greeted by a volunteer staff member who *admits* them to the hospital and provides them with a hospital wristband with their name and QR code that is used to interact with the exhibits and access the surveys. The exhibits and activities are divided into six zones (over three sections) to provide an interactive educational exhibition on mental health care. They include relics, demonstrations, animated and interactive displays, and a vivid virtual reality simulation of psychiatric symptoms. When visitors move through each zone, they learn about and experience how mental health care has changed over the years.

Visits at Mind Space begin at the “Past” section as visitors are guided through various exhibitions to understand how old-fashioned and stereotyped ideas about mental health conditions and their restrictive treatments are misplaced in the modern world, and how some archaic perceptions about mental health contribute toward stigmatized views in today’s society. Myths about mental health conditions are also debunked, and the benefits and nature of modern psychiatric treatment are explored as they proceed to the “Present” section of the exhibition. The “Present” section of Mind Space provides information about neurobiological and biopsychosocial views on different mental health conditions and also includes an interactive learning experience where visitors can personally experience hallucinations through virtual reality and simulations in order to build their empathy with people who experience symptoms of severe mental health conditions. The virtual reality contents are based on the actual experiences of the people with lived experience of mental illness that volunteer at Mind Space and this is revealed in the debriefing session at the end of the visit. The final section of the exhibition focusses on the “Future”, which aims to help visitors explore effective ways to improve their own mental health from a psychosocial perspective. The exhibition ends with a debriefing session with people who have lived experience of mental illness. The debriefing aims to further drive awareness of mental health conditions, facilitate direct contact with someone who has personal experience of recovering from mental illness, and help visitors contextualize the experiential, simulation, and interactive activities at Mind Space.

Mind Space was designed to provide an inclusive space that is suitable for all members of the public to enhance their understanding of and improve their attitudes toward mental disorders and seeking professional psychological support. Mind Space visitors were able to engage in a guided tour or could move through the exhibition unguided. The planned duration of the Mind Space visit was 3 hours (full visit). However, due to visitor restrictions imposed by COVID-19, some visitors were offered a shortened visit (less than 3 hours). Irrespective of the type of visit, all visitors met with volunteers with lived experience of mental illness at the end of the exhibition to provide a debriefing and to talk through their own recovery journeys and treatment experiences.

### Participants

All visitors to Mind Space aged 13 years old and above were invited to participate in this evaluation. Participants needed to be able to understand and read Chinese and/or English and provide informed consent (participants under 18 provided assent with the consent of their parent or legal guardian). No exclusion criteria were applied.

### Data collection

Visitors were asked to complete a range of questionnaires and provide demographic information online when registering to visit Mind Space and re-complete these questionnaires immediately after the debriefing sessions at the end of their visit. Each visitor was allocated a unique study ID number upon registration and used the same ID to log onto the online survey system to complete the post-visit questionnaires. All survey tools were completed electronically, anonymized to maintain confidentiality, and were managed by the Hong Kong Hospital Authority staff. Data were extracted from the online survey system in February 2022 for analysis.

### Outcome measures

Visitors completed a demographic questionnaire consisting of 10 items (i.e. sex, age, employment status, visitor type, highest academic qualification, accommodation, living situation, household income, marital status, types of previous personal contact with someone with a mental illness) during visit registration. During visit registration and at post-visit follow-up, visitors also completed the following survey instruments:

The Attitudes and Understanding Towards Mental Disorders (AUM) scale (44) was used to measure visitors’ beliefs and attitudes toward mental illness. This questionnaire consists of 15 statements related to stigma and discrimination associated with mental illnesses. Responses were captured on a 5-point Likert scale ranging from “Strongly Agree” to “Strongly Disagree.” A total summed score was calculated after reverse scoring of 3 items (3, 5, 7); higher scores indicated more negative attitudes toward mental illnesses. The Chinese version has good psychometric properties and has been effectively used with the general public in Hong Kong (44).

The Attitudes Toward Seeking Professional Psychological Help questionnaire (45) is a 29-item instrument that measures the propensity for seeking professional help for mental disorders. Item responses are based on a 4-point Likert scale (0 = strongly disagree, 1 = disagree, 2 = agree, and 3 = strongly agree). After the reverse scoring of 18 items, a total score is generated with a possible range of 0 to 87, with higher scores indicating higher propensity to seek help. A Chinese version of the original instrument (46) was used and demonstrated satisfactory psychometric properties, including reliability and validity, in ethnic Chinese people.

Visitors who self-identified as health and social care professionals also completed the Stereotype and Restriction Scale (SRS) to assess their attitudes toward people with mental health conditions (47). For stereotype assessment, the professionals were asked to indicate the extent to which they viewed patients with mental health conditions to be different from the general public (i.e. stereotyping) on a 5-point Likert scale (1 = ‘much less’ to 5 = ‘much more’) across 12 characteristics. For restriction assessment, they were asked to indicate the extent to which they agree on certain restrictions of behaviors or individual rights for people with mental health conditions on a 4-point Likert scale in four questions. Internal consistencies of the SRS were satisfactory among health professionals in Switzerland (47) and Hong Kong (48).

### Statistical analysis

The survey data were analyzed using SPSS version 23. Very minimal missing data were observed (<0.1% of data points). To manage the missing data in pre-visit surveys, mean/mode substation was employed. To manage missing data in post-visit surveys, the last observation carried forward method was adopted. Statistical significance was set at alpha < 0.05. Descriptive statistics were used to contextualize the demographic characteristics of the visitors and their baseline scores. Pearson correlations were calculated to examine the relationships between the outcome variables. Independent samples t-tests and one-way ANOVA were used to assess the relationships between demographic factors and the outcome variables (i.e., AUM & ATSPPH) at baseline. Additionally, to test our hypothesis that selected sociodemographic characteristics would be significantly associated with the study outcome measures two separate multiple linear regression models were built to identify the predictors of the AUM and ATSPPH based on the selected demographic factor using p<0.25 in bivariate analyses as a cut-off value for variable selection (49). The selected demographic factors were entered into the regression models in one block. Last, to test the study’s main hypothesis paired samples t-tests were used to examine differences in AUM and ATSPPH scores at pretest versus posttest. We also calculated Cohen’s d to measure the effect sizes; effect sizes were interpreted as small (d = 0.2), medium (d = 0.5), or large (d = 0.8) by convention (50).

## Results

A total of 1044 visitors registered to attend the Mind Space mental health experience exhibition through the online system from September 2019 to 31 December 2021. Of the registered visitors, 474 provided survey data before and/or after the visit. Of 474 visitors who provided survey data, 92 were omitted from the present analysis (16 registered but did not attend; 76 completed the post-visit survey only). The final dataset consisted of 382 participants who completed the pre-test, among which 146 also completed the post-test.

### Visitor characteristics

**Table 1** summarizes visitors’ demographic information for the full sample and those who completed pre-visit survey only versus those completing both pre- and post-visit surveys. Of the 382 visitors who provided baseline data, the majority were females (61.3%), healthcare professionals (82.2%), below age 30 (52.1%), and employed full-time (52.9%). More than half (56.8%) had personal contact with someone with a mental health condition. The largest proportion of visitors were from health or social care services (36.1%), followed by a university (21.2%), and then school/college (18.6%).

**Table 1:** Demographic characteristics of visitors to Mind Space.

	Full sample (n=382)	Pre- & Post-test (n=146)	Pre-test only (n=236)
Sex (n, %)			
Male	148 (38.7)	54 (37.0)	94 (39.8)
Female	234 (61.3)	92 (63.0)	142 (60.2)
Age Group (n, %)			
Below 30	199 (52.1)	86 (58.9)	113 (47.9)
31-50	118 (30.9)	41 (28.1)	77 (32.6)
51 or above	65 (17.0)	19 (13.0)	46 (19.5)
Employment Status (n, %)			
Employed (Full time)	202 (52.9)	71 (48.6)	131 (55.5)
Student	155 (40.6)	63 (43.2)	92 (39.0)
Unemployed or Underemployed	25 (6.5)	12 (8.2)	13 (5.5)
Highest Academic Qualification (n, %)*			
Below University	89 (23.3)	46 (31.5)	43 (18.2)
University or above	293 (76.7)	100 (68.5)	193 (81.8)
Accommodation (n, %)			
Privately Owned	240 (62.8)	84 (57.5)	156 (66.1)
Other	142 (37.2)	62 (42.5)	80 (33.9)
Living Situation (n, %)*			
Alone	43 (11.3)	28 (19.2)	15 (6.4)
With Family	339 (88.7)	118 (80.8)	221 (93.6)
Have Children (n, %)*			
Yes	79 (20.7)	21 (14.4)	58 (24.6)
No	303 (79.3)	125 (85.6)	178 (75.4)
Monthly household income in HKD (n, %)*			
25000 or below	119 (31.2)	59 (40.4)	60 (25.4)
25001 - 70000	163 (42.7)	59 (40.4)	104 (44.1)
70001 or above	100 (26.2)	28 (19.2)	72 (30.5)
Marital Status (n, %)*			
Single or divorced	256 (67.0)	112 (76.7)	144 (61.0)
Married or cohabited	126 (33.0)	34 (23.3)	92 (39.0)
Personal contact with a mental health condition (n, %)			
Yes	217 (56.8)	91 (62.3)	126 (53.4)
No	165 (43.2)	55 (37.7)	110 (46.6)
Personal contact with a mental health condition (n, %)			
Family member	42 (11.0)	20 (13.7)	22 (9.3)
Friend	100 (26.2)	45 (30.8)	55 (23.3)
Work colleague	41 (10.7)	17 11.6)	24 (10.2)
Neighbor	13 (3.4)	8 (5.5)	5 (2.1)
Classmates	20 (5.2)	6 (4.1)	14 (5.93)
Others	76 (19.9)	30 (20.5)	46 (19.5)
Health Care Professional (%)			
Yes	314 (82.2)	125 (85.6)	189 (80.1)
No	68 (17.8)	21 (14.4)	47 (19.9)
Group Tour (%)			
Yes	318 (83.2)	136 (93.2)	182 (77.1)
No	64 (16.8)	10 (6.8)	54 (22.9)
Visitor Type (%)			
Business	0 (0)	0 (0)	0 (0)
School/College	71 (18.6)	41 (28.1)	30 (12.7)
University	81 (21.2)	31 (21.2)	50 (21.2)
Government/Civil Service	14 (3.7)	4 (2.7)	10 (4.2)
NGO	2 (0.5)	2 (1.4)	0 (0)
Health or Social Care Service	138 (36.1)	43 (29.5)	95 (40.3)
Other	25 (6.5)	21 (14.4)	4 (1.7)
Visit Time (n, %)			
<3 hours	204 (53.4)	48 (32.9)	156 (66.1)
3 hours	178 (46.6)	98 (67.1)	80 (33.9)
Guided Tour (n, %)			
Yes	210 (55.0)	106 (72.6)	104 (44.1)
No	172 (45.0)	40 (27.4)	132 (55.9)
AUM mean score (SD)*	34.23 (6.70)	35.45 (6.92)	33.48 (6.47)
ATSPPH mean score (SD)	51.86 (8.22)	51.08 (7.07)	52.34 (8.83)
SRS-S mean score (SD)	39.99 (4.94)	40.09 (5.02)	39.93 (4.90)
SRS-R mean score (SD)	9.65 (1.45)	9.63 (1.37)	9.67 (1.51)

*Significant difference at p<0.05 between those completed pre-test only versus those completed both pre- and post-tests

Among all visitors included in this analysis (n=382), 46.6% attended the full visit (3 hours). Over half of the visitors (55%) participated in a guided tour. Their baseline mean scores on beliefs and attitudes toward mental illness (AUM) was 34.23 (SD = 6.70), and tendency to seek help for mental illnesses (ATSPPH) was 51.86 (SD = 8.22). For the attitudes of health care professionals toward people with mental illness (SRS), the mean score of the two sub-scales (SRS-S and SRS-R) at baseline were 39.99 (SD = 4.94) and 9.65 (SD = 1.45), respectively (See **Table 1**). AUM score was moderately negatively correlated to ATSPPH score (r= -.640, p<.001), but weakly positively correlated to SRS-R score (r=.207, p<.001). A weak negative correlation was observed between ATSPPH score and SRS-R score (r=-.217, p<.001). A weak positive correlation between SRS-S and SRS-R scores (r=.169, p=.003) was also observed.

### Demographic predictors of AUM and ATSPPH at baseline

Bivariate associations between each demographic characteristic with AUM and ATSPPH scores are described in **Table 2**.

**Table 2:** Demographic differences in outcome variables at baseline (n=382).

Variables	AUM			ATSPPH		
	M	SD	*p*	M	SD	*p*
Sex						
Female	34.72	6.636	ns	51.16	7.308	.048
Male	33.46	6.754		52.97	9.396	
Highest Academic Qualification						
Below University	38.25	6.377	<.001	48.76	6.350	<.001
University or above	33.01	6.319		52.80	8.492	
Accommodation						
Privately Owned	33.58	6.712	.014	52.63	8.729	.018
Other	35.32	6.563		50.56	7.111	
Marital Status						
Single/divorced	34.60	7.032	ns	51.34	8.511	ns
Married/cohabited	33.48	5.929		52.91	7.504	
Healthcare Professional						
Yes	33.73	6.613	.002	52.40	8.301	.005
No	36.56	6.663		49.35	7.364	
Personal contact with mental health conditions						
Yes	33.43	6.704	.007	53.13	8.225	<.001
No	35.28	6.569		50.18	7.921	
Living Situation						
Alone	34.12	6.984	ns	53.21	8.064	ns
With Family	34.24	6.675		51.69	8.231	
Have Children						
Yes	33.61	5.988	ns	52.16	7.702	ns
No	34.39	6.875		51.78	8.355	
Age Group						
Below 30	34.82	7.123	ns	51.13	8.507	ns
31-50	33.43	6.176		52.30	7.651	
51 or above	33.86	6.182		53.31	8.174	
Employment Status						
Employed (Full Time)	33.00	6.152	<.001	53.44	7.978	<.001
Student	35.15	6.965		49.93	8.279	
Unemployed/Underemployed	38.40	6.958		51.04	7.271	
Monthly Household Income in HKD						
25000 or below	36.32	6.862	<.001	49.56	6.682	<.001
25001 - 70000	33.67	6.491		52.07	8.435	
70001 or above	32.66	6.285		54.24	8.831	

ns, not significant.

Results of two separate multiple linear regression models regressing demographic variables on AUM or ATSPPH scores are presented in **Table 3**. Findings showed that demographic characteristics significantly predicted AUM scores, but only accounted for 14.8% of the model variance (**r**² = 0.148, F(12, 369) = 5.32, p<.001). Specifically, higher levels of education (β = -.268, p<.001) and personal contact with mental illness (β = -.117, p=.018) predicted lower scores on the AUM, whereas unemployment/underemployment predicted higher AUM scores (β = .117, p=.035). Demographic characteristics also significantly predicted ATSPPH and accounted for 11.2% of the model variance (**r**² = 0.112, F(12, 369) = 3.88, p<.001; **Table 3**). Compared to those employed full time, being a student (β = -.155, p=.037) predicted lower propensity for seeking professional psychological help (ATSPPH), while higher education level (β = .125, p=.031) and personal contact with mental illness (β = 147, p=.004) predicted higher scores in ATSPPH.

**Table 3:** Demographic predictors of outcome variables at baseline (n=382).

	B	Beta		t		*p*
Model 1: AUM						
Age (ref: below 30)						
31-50	.473	.033		.49		.628
51 or above	-.512	-.029		-.42		.678
Employment Status (ref: fulltime employment)						
Student	.651	.048		.66		.512
Unemployed or Underemployed	3.176	.117		2.11		.035
Monthly Household Income (ref: below 25000)						
25001-70000	-.454	-.034		-.48		.632
70001 or above	-.433	-.028		-.35		.726
Sex (ref: Female)						
Male	.087	.006		.13		.900
Health care professional (ref: No)						
Yes	-1.277	-.073		-1.44		.150
Accommodation (ref: Privately owned)						
Other	.142	.010		.19		.848
Highest Academic Qualification (ref: below university)						
University or above	-4.244	-.268		-4.72		<.001
Personal Contact with someone with a mental health condition (ref: No)						
Yes	-1.587	-.117		-2.37		.018
Marital Status (ref: single/divorced)						
Married/cohabited	-.152	-.011		-.17		.864
Model 2: ATSPPH						
Age (ref: below 30)						
31-50	-2.165	-.122		-1.77		.077
51 or above	-.797	-.037		-0.52		.605
Employment Status (ref: fulltime employment)						
Student	-2.595	-.155		-2.09		.037
Unemployed or Underemployed	-.556	-.017		-0.30		.767
Monthly Household Income (ref: below 25000)						
25001-70000	.828	.050		.70		.486
70001 or above	2.110	.113		1.36		.174
Sex (ref: Female)						
Male	.774	.046		.89		.375
Health care professional (ref: No)						
Yes	1.421	.066		1.28		.200
Accommodation (ref: privately owned)						
Other	-.559	-.033		-.60		.548
Highest Academic Qualification (ref: below university)						
University or above	2.428	.125		2.16		.031
Personal Contact with someone with a mental health condition (ref: No)						
Yes	2.439	.147		2.91		.004
Marital Status (ref: single/divorced)						
Married/cohabited	-.474	-.027		-.43		.670

### Changes in AUM and ATSPPH after visiting Mind Space

Among the 382 participants who completed the baseline survey, 146 participants (38.22%) also completed the questionnaires after attending Mind Space. An ad-hoc analysis revealed mean AUM scores was higher among those who completed both pre and posttests (M=35.45, SD=6.92) compared with those who completed the pretest only (M=33.48, SD=6.47). No significant difference was found in ATSPPH and SRS scores among those who did versus did not provide post-visit data.

**Table 4** shows the changes in the variables among 146 participants who provided pre-test and post-test data. The results showed significant decreases in visitors’ negative beliefs and attitudes toward mental illness (t=4.36, p = <.001; d = .361) and a significant increase in propensity for seeking professional help for mental disorders (t=-5.20, p<.001; d = -.430). Among health care professionals, significant changes in the stereotype (t=4.71, p=<.001; d = .421) and restrictions (t=2.29, p=.024; d = .205) SRS subscales scores were also observed.

**Table 4:** Differences in outcome variables before and after the visit.

	Pre-test	Post-test	t	*p*
	Mean (SD)	Mean (SD)		
AUM, (n=146)	35.45 (6.92)	33.18 (7.00)	4.36	<.001
ATSPPH, (n=146)	51.08 (7.07)	54.10 (8.94)	-5.20	<.001
SRS - S (n=125)	40.09 (5.02)	38.11 (5.53)	4.71	<.001
SRS - R (n=125)	9.63 (1.37)	9.34 (1.50)	2.29	.024

## Discussion

The present evaluation provides preliminary findings on the effectiveness of Mind Space – the first mental health learning experience to improve mental health stigma for members of the Hong Kong public from different backgrounds using a variety of educational approaches. Our data showed that visiting Mind Space was associated with a significant reduction in negative beliefs/attitudes about mental health conditions, and improvements in visitors’ propensity to seek professional psychological support. Among health care professionals, stereotyping attitudes and perceived needs to apply restrictions to persons with mental health conditions also decreased significantly immediately following the visit.

Although anti-stigma mental health interventions are widespread globally, interventions vary in their strategies and effectiveness. Previous reviews have highlighted that engaging in social contact with individuals with mental health conditions is the most effective way to improve stigma-related attitudes, yet the results in around half of the studies only sustained medium-term effects (37, 38). Thus, a single-component approach may not be the most efficient way to improve stigma-related attitudes. Our findings suggest that the multi-component approach of Mind Space (i.e. providing direct contact with individuals with mental illness, exhibits, virtual reality simulations, and experiential and interactive activities) effectively reduces mental health stigma among the general public and healthcare professionals immediately after the visit. While longer-term follow-ups are needed to ascertain whether these effects are sustained over time, the results provides preliminary support for integrating a multi-component approach in anti-stigma campaigns and interventions.

The current study’s findings also build upon previous results and recommendations generated from multi-component anti-stigma public events. For example, an evaluation of a national, two-week mental health arts festival for the general public in Scotland (51) showed overall improvements in attendees’ stigmatized belief about people with mental health conditions, but also an increase in the belief that people with mental health conditions are dangerous. When the ten individual events at the festival were analyzed separately, events that engaged dialogue with and highlighted positive representations of people with mental health challenges resulted in significant decreases in stigma, whereas an event showing a documentary film with images of violence and unpredictability without contextualization accounted for the significant increases in perceptions of dangerousness. Although Mind Space included exhibits regarding dangerousness and a virtual reality simulation of psychotic symptoms that may be potentially confronting, visitors received debriefing with a person with experience with mental health conditions immediately after the tour to support contextualization of those activities.

This evaluation also presented an opportunity to examine whether and how visitor characteristics associated with attitudes toward mental health conditions and propensity to seek professional mental health care. Overall, demographic characteristics alone appeared to be weak predictors, accounting for 15% of variance in explaining attitudes and understanding toward mental disorders and 11% of variance in explaining propensity toward seeking professional psychological help. However, our findings corroborate with existing evidence demonstrating that higher socioeconomic status (i.e. education and full-time employment) and having personal contact with someone with a mental health conditions predicted better attitudes and knowledge toward mental disorders and higher propensity to seek mental health care in Hong Kong (28–32).

### Study limitations

Several limitations are noted. First, the evaluation was conducted during COVID-19 and visits to Mind Space were severely restricted. Therefore, the present analysis only serves as a preliminary evaluation of Mind Space in improving mental health stigma. Second, visitors to Mind Space were self-selected to participate, and we note that vast majority of the visitors were healthcare professionals or had personal contact with someone with a mental health condition. Further, nearly 62% of the participants did not compete the post-visit survey, and attitudes/beliefs toward mental health conditions significantly differed between those who provided pre- and post-visit data as compared with those who only completed the pre-visit survey. Therefore, our results should be interpreted with caution and may not be fully generalizable to the Hong Kong public or future attendees. All outcome measures were also self-completed, which may have introduced social desirability bias and hence inflated the preliminary effects of attending the exhibition on the study outcomes. Finally, the lack of a control group in this study also precludes confident estimates of intervention effects on the study outcomes.

### Implications for practice, education, research and health policies

As Mind Space becomes more accessible to the public, continued evaluation using longitudinal designs and longer follow-ups are warranted to provide a clearer assessment of whether effects are sustained over time. Continued evaluation of Mind Space should incorporate strategies to promote post-visit survey completion because over half of visitors do not complete the post-visit survey. Inclusion of a control group and additional data sources (e.g. clinical data, visitor engagement, fidelity checking of tours by different guides) is also recommended in future evaluations to provide a more rigorous assessment of Mind Space’s effectiveness. Further, although we identified visitor characteristics associated with mental health stigma that corroborates with prior local studies, future investigations should include larger and more representative samples of the Hong Kong population so that the effectiveness of Mind Space in subgroups of citizens may be examined.

Given our positive findings, the importance of providing personal contact and a positive representation of people with mental health conditions in anti-stigma interventions is underscored. They also demonstrate that using a multi-component approach, including direct contact with people with lived experience of a mental health conditions and incorporating activities that are interactive and experiential in nature, have potential to reduce mental health stigma in the general public and health care professionals alike. Anti-stigma campaigns and interventions for the general public should therefore include more personable, interactive, and experiential portrayals or demonstrations of what it is like to experience mental distress. Our findings also highlight the need to target mental health education and promotion efforts among people with lower socioeconomic status in future mental health policy planning.

## Conclusion

The present evaluation study shows that higher socioeconomic status (i.e., education and full-time employment) and having personal contact with someone with a mental health conditions predicted more positive attitudes toward mental health conditions among the public and healthcare professionals in Hong Kong. This finding highlights the need for mental health education and promotion for people with lower socioeconomic status and the importance of having personal contact with someone with a mental health conditions in reducing stigmatizing attitudes. The present study also provided preliminary evidence that a multi-component approach, including direct contact with persons with experience of mental health conditions and immersive and interactive experiences, at a mental health exhibition can improve mental health stigma, thus suggesting Mind Space may be a valuable and transferrable model for public mental health education. Future longitudinal evaluations should include larger and more representative samples, with additions of control groups and other relevant exogenous measures, to provide a clearer assessment of the generalizability and sustained effectiveness of Mind Space.

## Data availability statement

The raw data supporting the conclusions of this article will be made available by the authors, without undue reservation.

## Ethics statement

The studies involving humans were approved by The Hong Kong Polytechnic University Ethics Committee and the Hong Kong Hospital Authority Cluster Clinical Research Ethics Committee. The studies were conducted in accordance with the local legislation and institutional requirements. The participants provided their written informed consent to participate in this study.

## Author contributions

GH: Writing – review & editing, Writing – original draft, Investigation, Funding acquisition, Formal analysis, Conceptualization. JM: Writing – review & editing, Project administration, Methodology, Investigation, Funding acquisition, Data curation, Conceptualization. RW: Writing – review & editing, Project administration, Methodology, Investigation, Funding acquisition, Formal analysis, Data curation, Conceptualization. WC: Writing – review & editing, Supervision, Methodology, Investigation, Funding acquisition, Conceptualization. LW: Writing – review & editing, Project administration, Formal analysis, Data curation. DB: Writing – review & editing, Writing – original draft, Supervision, Project administration, Investigation, Funding acquisition, Data curation, Conceptualization.
